# Impact of Irradiation on Vector Competence of *Aedes aegypti* and *Aedes albopictus* (Diptera: Culicidae) for Dengue and Chikungunya Viruses

**DOI:** 10.3389/fbioe.2022.876400

**Published:** 2022-06-03

**Authors:** Fabrizio Balestrino, Jérémy Bouyer, Marc J. B. Vreysen, Eva Veronesi

**Affiliations:** ^1^ National Centre for Vector Entomology, Vetsuisse Faculty, Institute of Parasitology, University of Zürich, Zürich, Switzerland; ^2^ Centro Agricoltura Ambiente “G. Nicoli”, Sanitary Entomology and Zoology Department, Crevalcore, Italy; ^3^ CIRAD, UMR ASTRE CIRAD-INRA « Animals, Health, Territories, Risks and Ecosystems », Montpellier, France; ^4^ FAO/IAEA Insect Pest Control Laboratory (IPCL), FAO/IAEA Joint Division of Nuclear Techniques in Food and Agriculture (NAFA), FAO/IAEA Agriculture and Biotechnology Laboratories, Vienna, Austria; ^5^ Laboratory of Applied Microbiology, Department of Environment, University of Applied Sciences and Arts of Southern Switzerland (SUPSI), Bellinzona, Switzerland

**Keywords:** sterile insect technique, dissemination, transmission, arbovirus, RRT-PCR

## Abstract

Effective control strategies against arthropod disease vectors are amongst the most powerful tools to prevent the spread of vector-borne diseases. The sterile insect technique (SIT) is an effective and sustainable autocidal control method that has recently shown effective population suppression against different *Aedes* vector species worldwide. The SIT approach for mosquito vectors requires the release of radio-sterilized male mosquitoes only, but currently available sex separation techniques cannot ensure the complete elimination of females resulting in short-term risk of increased biting rate and arboviral disease transmission. In this study, we compared for the first time the transmission of dengue and chikungunya viruses in *Aedes aegypti* and *Aedes albopictus* females exposed as pupae to an irradiation dose of 40 Gy. Females of both species were fed on blood spiked with either dengue or chikungunya viruses, and body parts were tested for virus presence by real-time RT-PCR at different time points. No differences were observed in the dissemination efficiency of the dengue virus in irradiated and unirradiated *Ae. albopictus* and *Ae. aegypti* mosquitoes. The dissemination of the chikungunya virus was higher in *Ae. albopictus* than in *Ae. Aegypti*, and irradiation increased the virus load in both species. However, we did not observe differences in the transmission efficiency for chikungunya (100%) and dengue (8–27%) between mosquito species, and irradiation did not impact transmissibility. Further implications of these results on the epidemiology of vector-borne diseases in the field are discussed.

## Introduction

Despite control measures applied worldwide over decades, mosquito-borne diseases continue to pose a constant threat to human and animal health. Globalization and climate change are resulting in the increased movement of mosquitoes and introductions and establishment of mosquito populations in areas where they could not survive before. This has contributed to a resurgence of important known diseases such as dengue fever, caused by the dengue virus (DENV), and new viruses which only recently have demonstrated their enormous pathogenic potential, such as the virus responsible for the Zika disease (ZIKV). *Aedes albopictus* and *Aedes aegypti* are considered the two most important mosquito vectors responsible for transmitting dangerous arboviruses circulating in tropical and temperate areas and that have the potential of imposing a significant global disease burden on half of the world’s population. Mosquitoes are recognized as the most invasive and deadly animal species in the world, and many of these species have expanded their distribution in all continents primarily through human-mediated transportation, despite attempts to reduce their density and prevent their establishment (Benedict et al., 2007; Paupy et al., 2009; Medlock et al., 2012; Schaffner et al., 2013; Schaffner et al., 2014). The increased and rapid development of the resistance of mosquitoes against existing and newly developed insecticides, the paucity of specific drugs or the lack of the development of new drugs, and the absence of effective vaccines against most of these arboviruses have stimulated the evaluation of alternative, sustainable, and effective mosquito control methods to successfully reduce the density and distribution of these important sanitary pests (Medlock et al., 2012).

The application of effective vector control tactics remains the main strategy for the management of many vector-borne diseases and the only approach available to protect populations against this nuisance (Wilson et al., 2020). The sterile insect technique (SIT) is an autocidal pest control method that requires the area-wide inundative releases of sterile insects (FAO, 2005) to induce sterility in the native female pest population. Consequently, the reproduction rate of the target population declines, resulting in a reduced density of the field population with each generation. It is, therefore, a type of “birth control” as wild female insects of the pest population do not reproduce when they are inseminated by the released sterilized males. In this type of autocidal control, sequential releases of sterilized insects in adequate sterile to wild male overflooding ratios lead to vector suppression, and hence, to the containment of the diseases these vectors transmit (Dame et al., 2009; IAEA et al., 2012; Bellini et al., 2013; Dyck et al., 2021). The SIT has been used all over the world as a part of area-wide integrated pest management (AW-IPM) programs over the past 70 years to successfully contain, reduce, eliminate, or prevent the establishment of insect pests of agricultural, veterinary, and medical importance (Dyck et al., 2021). In the last decade, several SIT pilot field trials have been implemented against several *Aedes* vector species worldwide with promising results (Oliva et al., 2021). However, many improvements in the mosquito “SIT package” are required to advance this control tactic toward a larger-scale operational level (Bouyer et al., 2020a; WHO/IAEA, 2020; Oliva et al., 2021). Sex separation remains one of the main challenges for the efficient application of the mosquito SIT. The release of sterile female mosquitoes has to be avoided at all costs as they could contribute to the transmission of these viruses. Although mechanical sex separation techniques of *Aedes* pupae do exist, a small percentage of females is still accidentally processed together with the males and then released in the field. It is not expected that the release of a small percentage of unwanted sterile females together with the males would have a significant impact on the efficiency of the SIT, but it might increase the biting rate, and hence, the risk of transmission of these arboviruses which will limit its applicability and more importantly, its political and ethical acceptability, especially in areas where these diseases are endemic (WHO/IAEA, 2020).

Virus transmission by arthropods is a complex process (vector competence) controlled by biological intrinsic barriers in the vectors, including barriers affecting virus amplification in the mid-gut cells, dissemination in the insect’s body fluid (hemolymph), and finally, infection and amplification in the salivary glands as the last stage before transmission to a new host. Ionizing radiation can influence the competence of different mosquitoes for pathogens and viruses through alterations of their immune response, and therefore, their ability to effectively transmit these arboviruses (Guissou et al., 2020). Radiation can also seriously alter symbiotic gut microbiota which is linked to the insect ecological fitness (Cai et al., 2018) and can affect immune responses that can influence vector competence for human pathogens (Dennison et al., 2014). Moreover, radiation can alter the feeding behavior and the survival rate of females under laboratory conditions (Cunningham et al., 2020). All of the aforementioned suggests the need for a more in-depth investigation of the effect of irradiation on vector competence in *Aedes* female mosquitoes.

The present study is the initial step of a more comprehensive investigation of the effect of irradiation (40 Gy) administered to *Ae. albopictus* and *Ae. aegypti* female pupae, on the transmission of dengue (DENV) and chikungunya viruses (CHIKV). We measure the viral RNA load of DENV and CHIKV in the saliva of irradiated and nonirradiated adult female mosquitoes as a means of transmission efficiency and transmission rate. Saliva samples were analyzed as individual females and in pools of eight samples/pool to compare the sensitivity of the test. Moreover, viral RNA load was also investigated in other body parts, to evaluate the propagation of the two viruses (dissemination efficiency) within the two species of irradiated and nonirradiated mosquitoes. The results will contribute to a better understanding of the implications and risks of unintentionally released irradiated *Aedes* females during AW-IPM programs that include an SIT component.

## Materials and Methods

### Mosquito Strains

The *Ae. albopictus* and *Ae. aegypti* strains used in this study were obtained from eggs collected in Rimini (Emilia-Romagna, Italy) and Juazeiro (Bahia, Brazil), respectively, by the Centro Agricoltura Ambiente CAA Italy and Moscamed Brasil, both IAEA collaborating centers for the development of the SIT package against *Aedes* vectors. Eggs were received from the FAO/IAEA Insect Pest Control Laboratory (IPCL, Seibersdorf, Austria) and reared under laboratory conditions (27 ± 1°C, 85 ± 5% RH, 16:8 h L:D photoperiod) at the National Centre for Vector Entomology of the University of Zürich (UZH, Zürich, Switzerland). Larvae obtained after standardized hatching procedures (Balestrino et al., 2010) were reared at a fixed larval density (2 larvae/mL) and fed with IAEA-BY liquid diet (3.0% w/v) at a mean daily dose of 0.5 mg/larvae (Balestrino et al., 2014) for the first four days of development. Pupae harvested on the sixth day from larval introduction were sexed under a stereomicroscope and aged at least 24 h before being subjected to irradiation treatments.

### Mosquito Irradiation

About 1,000 *Ae. albopictus* and *Ae. aegypti* female pupae were transferred into separated tissue culture flasks (type T75, 250 ml capacity) each containing 210 ml of deionized water for irradiation procedures. The quantity of water in the flask was used to standardize the pupal irradiation exposure during treatment by maintaining the floating position of the pupae at 3 cm from the flask’s bottom. Flasks were transported to the Department of Radiation Oncology, University Hospital of Zürich (USZ), where irradiation was carried out with a TrueBeam linear accelerator (TrueBeam^®^ STx, Varian Medical Systems, Palo Alto, CA) at 40 Gy (dose rate 6.2 Gy/min, photons energy 6.0 MV) [48]. Immediately after irradiation, the pupae were transported back to the UZH Insectary and placed in plastic cups (diameter 7 cm, height 8 cm) with about 150 ml of deionized water for emergence inside two separated polyester netting cages (32.5 × 32.5 × 32.5 cm) (BugDorm 43030F, MegaView Science Co., Ltd., Taichung, Taiwan). Each cage was provided with about 1,000 conspecific fertile males to assure the mating status of females and their optimal post-mating biting behavior. A 10% sucrose solution was supplied as a carbohydrate source and the cages were kept in a climate chamber under laboratory conditions (27°C with 85% RH and 16:8 h L:D photoperiod). In this preliminary study, we applied a dose of 40 Gy to 24–30 h old *Ae. aegypti* and *Ae. albopictus* pupae, shortly before adult emergence. This dose and time of treatment were selected as it resulted in male residual sterility of around 1% for both species with a low impact on sterile insect quality (Balestrino et al., 2010; Balestrino et al., 2017; Culbert et al., 2018).

### Viruses

Two viruses were used for mosquito oral inoculation: dengue type 2 virus strain (Vazeille et al., 1999) (DENV-2) from Bangkok and chikungunya strain 06.21 (Vazeille et al., 2007) (CHIKV) from La Réunion Island. Both viruses were amplified twice in C6/36 cell lines before their use for oral inoculation of mosquitoes. Briefly, C6/36 cells were grown in cell culture flasks (T25, 50 ml capacity) maintained in a Leibovitz L-15 medium supplemented with 1% antibiotics-antimycotics (penicillin, streptomycin, and amphotericin B), and fetal calf serum (FCS) at a concentration of 4 and 10% for CHIKV and DENV, respectively. Confluent C6/36 cells were inoculated with 100 µL of the original virus generating a C6/36 passage 1 (P1). All the flasks were incubated for three days (CHIKV) and five days (DENV) at 28°C with 5% CO_2_. After the incubation period, the supernatants were harvested and 200 µL inoculated into a new C6/36 cell flask (T75, 250 ml capacity) to generate a P2 passage at a multiplicity of infection (MOI) of 0.1. To confirm the infectivity of all passages, 10-fold serial dilutions of P1 and P2 supernatants were titrated on 96-well plates layered with C6/36 cells. Briefly, 96-well plates were seeded with 6.2 × 10^6^ cells/mL and incubated at 28°C and 5% CO_2_ for 24 h before their inoculation with the virus. For the inoculation, media were removed from all the well plates followed by inoculation of 50 µL/well of each ten-fold serial dilutions (using four replicates per dilution) and incubated for 1 h at 28°C and 5% CO_2_. After the incubation time, each well was overlaid with 150 µL of a premix 1:1 of sterile CMC (carboxymethylcellulose sodium salt, Sigma), 3.2% (water and 0.85% NaCl), and an L-15 medium supplemented with 10% FCS, and then the plates were sealed and incubated at 28°C with 5% CO_2_. On day three (CHIKV) or day five (DENV) post-incubation, the cells were fixed by adding 100 µL/well of formaldehyde 3.6% (in PBS) (without removal of the overlay) followed by an incubation period of 20 min at RT. After this time, the content from each well (medium and formaldehyde) was removed and the cells were rinsed three times with PBS. To detect viral foci, an immunoperoxidase assay was performed. Briefly, the wells were incubated in Triton (0.5% in PBS) for 5–15 min at RT followed by three PBS washes. The wells were incubated for 30–45 min at 37°C with the primary antibody at the appropriate concentration in PBS (1:200 for DENV and 1:1000 for CHIKV) followed by three PBS washes. The secondary antibody goat anti-mouse igG (H + L) (Alexa Fluor^®^ 488; ThermoFisher Scientific Inc., USA) was added (1:500 in PBS for both viruses) and incubated for 30 min at 37°C and the final three PBS washes were applied before examination of the fluorescence in each well by an indirect immunofluorescence assay (IFA). Tissue culture infectious dose (TCID_50_) of both viruses was calculated by the observation of fluorescence at each dilution series and the titers were calculated as the last dilution recorded positive at the 50% end-point and expressed as log_10_TCID_50_/mL which was later converted to log_10_ plaque-forming units/mL (log_10_ PFU/mL) (O`Reilly et al., 1994). Finally, a standard curve of the two viruses was generated by converting the viral RNA amount cycle threshold (Ct) values into PFU. Briefly, viral RNA from the supernatant of the second virus passages (DENV C6/36 P2 and CHIKV C6/36 P2) was extracted using a viral nucleic acid kit (Qiagen QIAmp viral RNA mini kit), and a real-time reverse transcriptase polymerase chain reaction (rRT-PCR) of ten-serial fold dilutions of viral RNA was performed (Mousson et al., 2010; Mousson et al., 2012). Finally, Ct values from each serial dilution were challenged toward the standard curves to infer the equivalent number of PFU/mL to the Ct values.

### Mosquito Infection

Infection and incubation of mosquitoes were carried out in the BSL3 laboratory of the Laboratory Animal Service Centre (LASC) at the UZH. Seven-day-old *Ae. aegypti* and *Ae. albopictus* irradiated females (F0), caged with conspecific fertile males, were deprived of sugar for 24 h before their exposure to heparinized sheep blood spiked with either CHIKV or DENV at a final concentration of 8.0 log_10_TCID_50_/mL (17.2 Ct) and 6.0 log_10_TCID_50_/mL (21.21 Ct), respectively. The virus-spiked blood was transferred to a Hemotek feeder (Hemotek Ltd., Lancashire, United Kingdom) layered with a membrane Parafilm M (Sigma-Aldrich, Buchs, Switzerland) and kept at a constant temperature of 36 °C during the whole feeding process. After 30 min of feeding, fully engorged females of both species were transferred into a netted cardboard pot (approximately 80 females/pot) with a 10% sucrose solution imbibed cotton pad on top of the net. All the pots were incubated in a climate chamber as described previously for 7 (CHIKV) or 14 (DENV) days post-inoculation (dpi). As a control, unirradiated mosquitoes from both species were exposed to CHIKV or DENV, incubated, and processed as irradiated mosquitoes. Freshly engorged *Ae. aegypti* and *Ae. albopictus* females for each irradiated and unirradiated group were collected at day 0 (day 0 females) and a sample of the infectious blood mixtures used for oral inoculation and processed for rRT-PCR to confirm infection and provide baseline data.

### Mosquito Dissection and Saliva Collection

After the incubation period, 100 surviving female mosquitoes from each treatment were processed. To collect saliva from live females, legs and wings were removed and stored in individual 1.5 ml Eppendorf tubes previously filled with 100 µL of DMEM supplemented with 1% antibiotics-antimycotics (penicillin, streptomycin, and amphotericin B) and 10% FCS. Females deprived of legs and wings were allocated on a flat surface and their proboscides were inserted into 5 µL glass capillary tubes filled with 10% FCS. Females were left in this position for 30 min after which the proboscides were removed from the capillary tubes and the contents of the capillary tube with the possibly spitted saliva flushed into a 1.5 ml Eppendorf tube filled with 400 µL of DMEM (10% FCS and 1% antibiotics-antimycotics as described previously). After saliva collection, the bodies of all females were dissected into 1) head and thorax and 2) abdomen, which were individually stored in separated 1.5 ml Eppendorf tubes at −80°C until further examination.

### Viral RNA Quantification

Dissemination of CHIKV and DENV was investigated by quantifying viral RNA (rRT-PCR) isolated from the legs and wings. These body parts from individual females were homogenized using the TissueLyser II instrument (TL) (Qiagen GmbH, Hilden, Germany). Briefly, one stainless steel bead (3 mm diameter) was added to each tube together with 100 µL of DMEM supplemented as described previously and processed with the TL at 25 Hz for 1 min. Additional 900 µL of DMEM was then added yielding a final volume of 1.0 ml. After centrifugation at 13,000 rpm for 5 min, 100 µL aliquots from eight samples were pooled (800 µL total volume/8 females). Viral RNA was extracted from each pool using the manual viral nucleic acid extraction kit (QIAmp viral RNA mini kit) described previously and rRT-PCR was performed as described elsewhere (Mousson et al., 2010; Mousson et al., 2012). Dissemination efficiency corresponds to the proportion of mosquito pools with infected legs and wings among tested ones.

To confirm the presence of viral RNA in saliva (transmissibility), the saliva of individual females that were positive in legs and wings (pools) were pooled and processed for viral RNA detection using the protocols described previously. Briefly, viral RNA was extracted from all females (*n* = 16) originated from two saliva positive pools (16 females per type of treatment [irradiated *vs*. unirradiated], vector species [*Ae. albopictus vs. Ae. aegypti*], and virus [CHIKV *vs*. DENV]). Saliva pools were chosen according to their Ct values: one pool with the highest Ct values and one with the lowest. Transmission efficiency corresponds to the proportion of mosquito pools with the virus in saliva among tested ones.

### Statistical Analysis

All statistical analyses were carried out using R statistical software (version 3.5.2). Pearson’s Chi-square test was applied to compare the dissemination and transmission efficiency for dengue and chikungunya viruses among irradiated and unirradiated *Ae. albopictus* and *Ae. aegypti* mosquito pools. The normality of the individual and pool data distributions was assessed using the two-sample Kolmogorov–Smirnov test. The data of the measurements of viral loads were normally distributed (*p* > 0.05). Concordance between individual values and their corresponding positive pools was determined by measuring the datasets through the Pearson correlation test. The viral loads observed were about 1–3 logs lower in mosquitoes infected with DENV than with CHIKV which were thus analyzed separately. A Gaussian linear mixed-effects model fit by maximum likelihood was used to analyze the virus titers (PFU/ml) separately for the two viruses (CHIKV *and* DENV) with mosquito species, irradiation, and their first-order interaction as fixed effects and the replicate as a random effect. The best model was identified by simplifying the complete one and considering the one with the lowest corrected Akaïke information criterion. The significance of the fixed effects was tested using the likelihood ratio test to compare models with or without these effects (Hurvich and Tsai, 1995; Burnham and Anderson, 2003).

## Results

### Mosquito Infection

Overall, 411 irradiated female *Ae. albopictus* and 427 *Ae. aegypti* were exposed to heparinized sheep blood spiked with DENV, and 296 irradiated female *Ae. albopictus* and 458 *Ae. aegypti* were exposed to CHIKV-spiked blood. The viral RNA amounts (Ct) measured in the infectious blood and in the day 0 females ranged between 25.7 and 35.0 Ct.

### Viral RNA Quantification

The virus inoculum (P0) used to generate P2 were 6.8 and 9.3 Log_10_ (PFU)/mL for DENV and CHIKV, corresponding to 19.26 and 13.44 Ct values for DENV and CHIKV, respectively. Standard curves of the DENV and the CHIKV (C6/36, P2) used in mosquito inoculations were calculated by linear regression of the log_10_ PFU/mL to the viral RNA amount (Ct) obtained from the rRT-PCRs of the corresponding dilution series. Standard curves showed a high correlation coefficient for the CHIKV (Log_10_ PFU = −0.3129*Ct + 13.468; *R* ^*2*^ = 0.999) and DENV (Log_10_ PFU = −0.3149*Ct + 12.723; *R* ^*2*^ = 0.952).

### Dissemination Efficiency and Viral Loads

**FIGURE 1:**
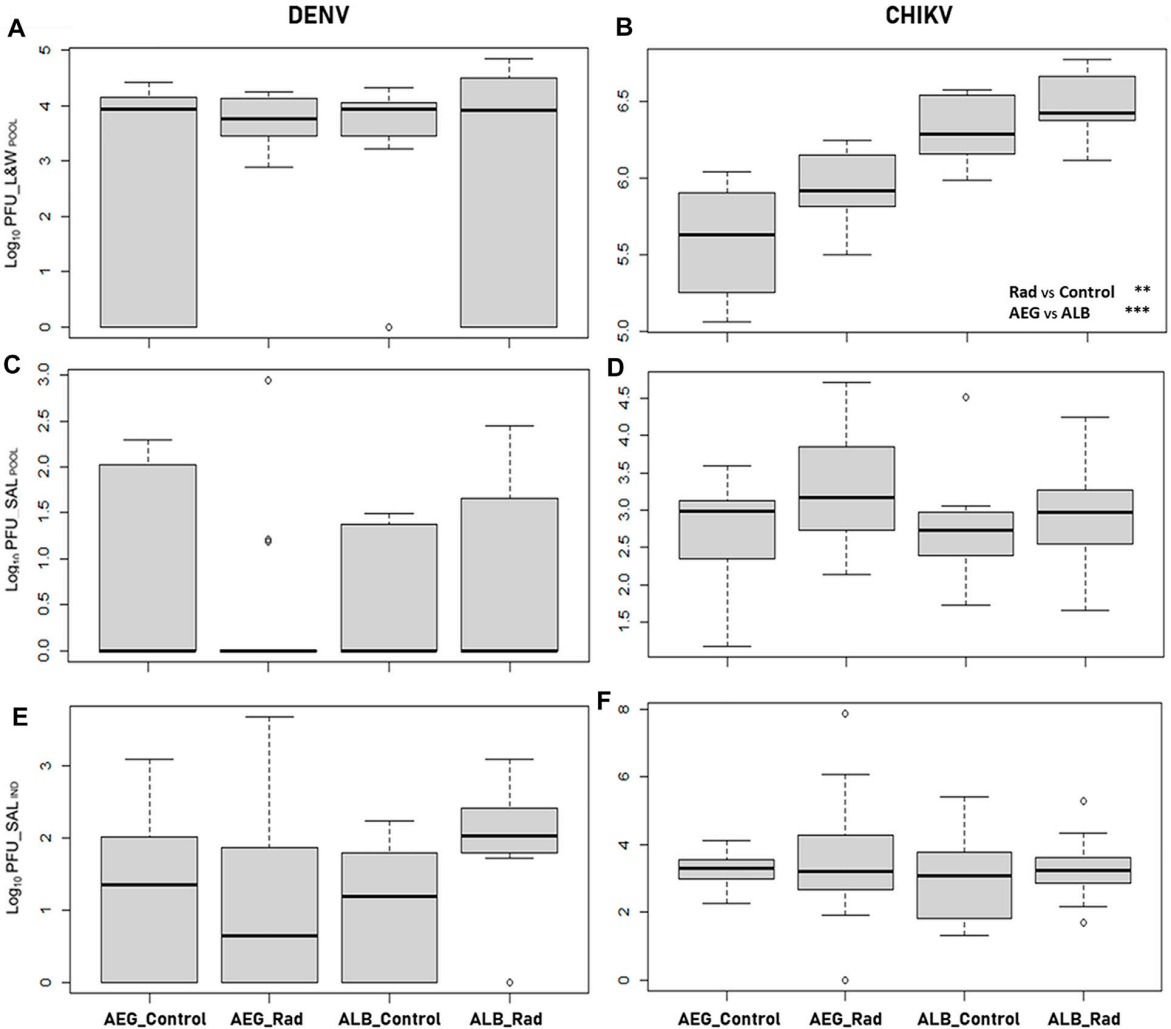
Boxplots of the viral load (log_10_ PFU) measured in pooled samples (POOL) of legs and wings (L&W) and saliva (SAL) from both irradiated (rad) and unirradiated (control) *Ae. aegypti* (AEG) and *Ae. albopictus* (ALB) mosquitoes infected with dengue (DENV, 14 dpi; or **(A,C)** chikungunya virus (CHIKV, 7 dpi; **(B,D)** Each pool sample consisted of homogenates of legs and wings and saliva from eight individual females. Viral load measured in saliva samples from individual females (IND) is also reported from both irradiated and not-irradiated *Ae. aegypti* and *Ae. albopictus* mosquitoes infected with (E) dengue or **(F)** chikungunya virus. There are no statistical differences in the different groups represented in the figure except for the significant effects of radiation (rad) and the species used (AEG or ALB) on the dissemination of chikungunya virus as shown in **(B)** and reported in Table 2. Asterisks represent statistical significance (**p < 0.01; ***p < 0.001).

Viral load measured in the pools of legs and wings from both irradiated and unirradiated mosquito species infected with CHIKV or DENV is shown in Table 1 (dissemination efficiency) and Figures 1A,B. All the pools of legs and wings from the mosquitoes infected with CHIKV were positive whereas for DENV, the percentage of positive pools ranged between 64 and 100% (Table 1). No differences were observed in the dissemination efficiency of irradiated and unirradiated mosquitoes infected with DENV at the pool level (χ^2^ = 6.3695, df = 3, *p* = 0.095; Table 1). In the case of DENV, we did not observe differences between the mosquito species (Figure 1A, likelihood ratio = 3.167, *p* = 0.205) and radiation had no effect on the viral load measured in both species (likelihood ratio = 3.249, *p* = 0.197) (Table 2). In the case of CHIKV, however, the viral load measured in *Ae. albopictus* was higher (Figure 1B, likelihood ratio = 10.578, *p* = 0.001) and irradiation increased the virus load in both species (likelihood ratio = 41.470, *p* < 10^–4^) (Table 2).

**TABLE 1:** Number and percentage of positive pools of legs and wings (dissemination efficiency) and saliva (transmission efficiency) from irradiated (rad) and unirradiated (control) *Ae. albopictus* and *Ae. aegypti* females orally fed with CHIKV and DENV after 7- or 14-days incubation, respectively. The range of log_10_ PFU measured (MIN—MAX) for legs and wings and saliva pools in both irradiated and unirradiated species infected with CHIKV or DENV is also reported. Each pool of legs and wings (L&W) and saliva (SAL) homogenates were derived from eight females.

				Dissemination efficiency (L&W)			Transmission efficiency (SAL)		
Virus	Status	Species	Pool (N)	PCR/+ (N)	PCR/+ (%)	Log_10_ PFU (min—max)	PCR/+ (N)	PCR/+ (%)	Log_10_ PFU (min—max)
CHIKV	Control	*Ae. albopictus*	8	8	100%	5.99—6.58	8	100%	1.73—4.51
CHIKV	Rad	*Ae. albopictus*	12	12	100%	6.11—6.04	12	100%	1.66—4.24
CHIKV	Control	*Ae. aegypti*	10	10	100%	5.06—6.77	10	100%	1.16—3.59
CHIKV	Rad	*Ae. aegypti*	12	12	100%	5.50—6.25	12	100%	2.13—4.70
DENV	Control	*Ae. albopictus*	8	7	88%	3.21—4.33	2	25%	0.00—1.48
DENV	Rad	*Ae. albopictus*	11	7	64%	0.00—4.84	3	27%	0.00—2.45
DENV	Control	*Ae. aegypti*	9	6	67%	0.00—4.41	2	22%	0.00—2.29
DENV	Rad	*Ae. aegypti*	13	13	100%	2.88—4.25	1	8%	0.00—2.94

**TABLE 2:** Fixed effects of linear mixed-effects models fit by maximum likelihood for the effect of the virus type (CHIKV and DENV) and radiation treatment (rad) on female viral loads measured in legs and wings and in the saliva on pooled (pool) and individual samples (individual). Values have been presented considering the two mosquito species together except for data on CHIKV presence in legs and wings (*) where the mosquito species and the radiation treatment were both kept in the best model.

Virus		Origin	Sample	Factors	Value	SE	df	t-value	*p* value
CHIKV	*	Legs and wings	Pool	Rad	0.261	0.078	28	3,342	0.002
	*	Legs and wings	Pool	*Ae. albopictus*	0.628	0.078	28	8,105	0.000
	—	Saliva	Pool	Rad	0.423	0.231	29	1,831	0.077
	—	Saliva	Individual	Rad	0.366	0.298	46	1,227	0.226
DENV	—	Legs and wings	Pool	Rad	0.296	0.523	27	0.566	0.576
	—	Saliva	Pool	Rad	−0.121	0.296	27	−0.408	0.687
	—	Saliva	Individual	Rad	0.420	0.283	41	1,483	0.146

### Transmission Efficiency and Viral Loads

Viral RNA presence was also confirmed among saliva collected from both irradiated and not-irradiated females on day 7 or day 14 after exposure to either CHIKV or DENV, respectively. Not all positive leg and wing pools were positive for viral RNA in the saliva. Nevertheless, 100% of saliva pools derived from *Ae. albopictus* and *Ae. aegypti* infected with CHIKV were positive, while only 8–27% of the saliva pools from females inoculated with DENV were positive with a lower PFU range (Table 1, transmission efficiency). Viral loads in pools of saliva from both irradiated and unirradiated mosquito species infected with CHIKV or DENV are shown in Table 1 (transmission efficiency) and Figures 1C,D. No differences were observed in the transmission rate of irradiated and unirradiated mosquitoes infected with DENV (χ^2^ = 1.2415, df = 3, *p* = 0.7431). For both viruses, we did not observe differences in the transmission rates between the mosquito species (likelihood ratio = 0.109, *p* = 0.742 for DENV and likelihood ratio = 0.273, *p* = 0.602 for CHIKV). Also, irradiation did not impact transmission rates (likelihood ratio = 0.172, *p* = 0.679 for DENV and Likelihood ratio = 3.493,315, *p* = 0.062 for CHIKV) (Table 2; Figures 1C,D).

A significant correlation was observed between the viral loads measured in the saliva pool samples and in the corresponding individual samples (*t* = 3.3424, df = 13; *p* = 0.0053; Cor estimated = 0.68). In DENV, the two mosquito species had a similar virus load (likelihood ratio = 4.343, *p* = 0.114) and irradiation only marginally increased transmission (likelihood ratio = 5.689, *p* = 0.058) (Table 2; Figures 1E,F). In CHIKV, no impact of mosquito species (likelihood ratio = 1.100, *p* = 0.294) nor irradiation (likelihood ratio = 1.522, *p* = 0.217) was observed (Table 2; Figures 1E,F).

## Discussion

This study demonstrated that the irradiation of female *Ae. aegypti* and *Ae. albopictus* as pupae with a dose of 40 Gy had no effect on the dissemination of DENV but increased the virus load of CHIKV in the mosquitoes’ bodies in both species. However, irradiation did not increase the transmission rate of both viruses in both mosquito species, although a marginal increase was detected at the individual level for DENV in *Ae. albopictus*. Both species are well-known vectors of these two viruses, with dissemination and transmission rates ranging between 33% and 87% for DENV (Vazeille et al., 2010; Guo et al., 2013; Gonçalves et al., 2014) and between 40% and 100% for CHIKV (Schuffenecker et al., 2006; Richards et al., 2010; Vega-Rúa et al., 2014), respectively. The highest virus dissemination of CHIKV was recorded in *Ae. albopictus*, regardless of irradiation treatment. The CHIKV strain used here is the natural mutant form with one amino-acid substitution (alanine → valine) in position 266 of the E1 glycoprotein (E1-A226V) (Schuffenecker et al., 2006). It was previously demonstrated that this strain is better adapted to *Ae. albopictus* than to *Ae. aegypti*, resulting in a high replication level and a shorter extrinsic incubation period (EIP) for both species, with infectious virus particles being detectable in the saliva as early as two dpi (Dubrulle et al., 2009). However, despite the larger dissemination efficiency observed in *Ae. albopictus* compared with *Ae. aegypti*, we did not observe a clear increase of CHIKV viral load between the saliva of the two species in either pools or individuals confirming the essential role of salivary glands in selecting viruses for efficient transmission (Vega-Rúa et al., 2014). Ct values of DENV-inoculated mosquitoes were generally higher than those of CHIKV-inoculated mosquitoes. Still, the initial viral dose of CHIKV (8.0 log10TCID50/mL) in the inoculum was 2 logs higher than the one used for DENV (6.0 log10TCID50/mL). However, DENV and CHIKV particles were well disseminated through the entire body with clear virus amplification between the initial intake of the infectious inoculum (day 0 females) and the end of the EIP (7 or 14 dpi for CHIKV and DENV, respectively) in both irradiated and unirradiated mosquitoes. Despite the high inoculum of CHIKV used for the females’ infections, it was still possible to observe a different pattern in the amplification of the virus at the dissemination stage (legs and wings), with a higher virus load among irradiated females of both species while irradiation treatment had no significant effect on viral load amplification in the salivary glands.

Exposure to ionizing radiation is the most used method to sterilize male insects in AW-IPM programs that include an SIT component (Bakri et al., 2005). Because irradiation may adversely influence the quality of the insect to be released through mutations in the somatic cells, it is essential to select the optimum radiation dose that effectively sterilizes the insect without adversely affecting the insect’s competitiveness (Bakri et al., 2005; Parker et al., 2021). Dose-dependent radiation damage to the insect’s mid-gut has been previously identified as the main factor affecting changes in the infection levels and in the alteration in the gut bacterial communities (Lauzon and Potter, 2012; Woruba et al., 2019; Guissou et al., 2020). The structural damages observed in the mid-gut tissues of irradiated flies lead to metabolic and physiological abnormalities (Lauzon and Potter, 2012) and could be related to the increased dissemination efficiency observed in irradiated female mosquitoes in this study. Microscopic observations on fruit flies irradiated at the dose used in the SIT suggested that radiation interfered with tissue formation and generated small breaks in the peritrophic membrane integrity, possibly affecting the gut functions, the passage of pathogens, and the overall quality and competitiveness in nature (Lauzon and Potter, 2012). Recently, it was reported that micro-perforations produced after consecutive blood feedings in the virus-impenetrable mid-gut basal lamina provided a mechanism for enhanced virus escape increasing dissemination and transmission efficiency for DENV and CHIKV in *Ae. albopictus* and *Ae. aegypti* (Armstrong et al., 2020). Although the impact of irradiation on mid-gut virus infection was not the topic of this work, our data indicate that an irradiation dose of 40 Gy delivered at the pupal stage increased the capability of the CHIKV variant E1-226V to overcome the intrinsic barriers of orally infected mosquitoes while we did not observe any increase in virus transmission in the irradiated mosquito for both species. The key role of the mid-gut barrier in CHIKV E1-226V dissemination through the mosquito body has been already demonstrated in *Ae. albopictus* in comparison with *Ae. aegypti* (Arias-Goeta et al., 2013) and possible damage in the mid-gut barriers following irradiation could therefore support the different CHIKV E1-226V dissemination efficiency observed in our trial between the two species.

The mid-gut constitutes an important component of the mosquito’s immune response defense against transmitted pathogens and it is the first barrier that viruses must cross to achieve a successful viral cycle (Janeh et al., 2017; Taracena et al., 2018). The effectiveness of an early mid-gut cellular renewal and the maintenance of mid-gut homeostasis during viral infection proved to be an important factor in the antiviral response of *Ae. aegypti* against DENV infections (Dennison et al., 2014; Taracena et al., 2018; Janeh et al., 2019). Because radiation-induced cellular damage could affect the viral permeability of the mid-gut membranes and delays the activation of the regenerative cellular process (Lauzon and Potter, 2012; Taracena et al., 2018), there may be a larger window of opportunity during which the mid-gut membrane of the irradiated mosquito becomes permissive for viral dissemination (Dong et al., 2017). The peculiar viral replication kinetics in the CHIKV strain (Merwaiss et al., 2021), with a shorter EIP, could have played a crucial role in increasing mid-gut escape and dissemination efficiency in irradiated mosquitoes in comparison with DENV in our trials. The marginal, not significant, increase of the viral load observed in *Ae. albopictus* females infected with DENV would be worth investigating in further studies.

In addition to the maintenance of an effective vector competence, the irradiated females accidentally released during an SIT campaign must effectively disperse, feed, and survive in the field to increase the risk of transmission of arboviral diseases. The mean survival rates observed during mark-release-recapture studies with irradiated and unirradiated *Aedes* males were similar with a mean lifespan estimated at two to eleven days (Muir and Kay, 1998; Bellini et al., 2010; Brady et al., 2013; Vavassori et al., 2019). As the more radiosensitive female mosquitoes have shorter survival than their male counterparts under laboratory conditions (Bond et al., 2019; Aldridge et al., 2020), further investigations will be required to estimate if irradiated females are capable of surviving the EIP to become infectious under field conditions. *Aedes albopictus* and *Ae. aegypti* female mosquitoes would need to survive six to twelve days in the field to acquire and effectively transmit arboviruses (Tjaden et al., 2013; Christofferson et al., 2014). Therefore, the average lifespan of irradiated *Aedes* females may not be sufficient to consider them effective vectors. Moreover, the reduced survival and blood-feeding frequency observed in irradiated *Aedes* females (Bond et al., 2019; Cunningham et al., 2020), and the increased mortality resulting from the ingestion of an infected blood meal (Maciel-de-Freitas et al., 2011; da Silveira et al., 2018), can have a significant impact on the overall vectorial capacity and transmission efficiency of irradiated *Aedes* females accidentally released in an operational *Aedes* SIT program. In the present study, only one time point of infection was investigated (days 7 and 14 for CHIKV and DENV, respectively) with relatively small sample sizes. Further studies would be therefore required to explore the temporal dynamics of viral dissemination and transmission in irradiated female mosquitoes fed with virus-spiked blood, that is, at daily intervals and with different radiation doses. This will reveal whether irradiation has an impact on the EIP as this could significantly influence the epidemiology of vector-borne diseases in the field. Moreover, the correlation between viral infection, mid-gut cell damages, and the alteration of the gut microbiota in relation to different radiation doses need to be further addressed to investigate the impact of radiation on female vector competence and male survival, flight ability, and field competitiveness.

This study showed that irradiation did not significantly alter the competence of irradiated female *Aedes* mosquitoes, illustrating the need for improved, more fail-proof sexing systems to reduce female contamination in released sterile males to enhance the acceptability of SIT programs and prevent any transmission risk.

## Data Availability

The original contributions presented in the study are included in the article/Supplementary Material; further inquiries can be directed to the corresponding author.

## References

[B1] Aldridge R. L. Kline J. Coburn J. M. Britch S. C. Boardman L. Hahn D. A. (2020). Gamma-irradiation Reduces Survivorship, Feeding Behavior, and Oviposition of Female *Aedes aegypti* . J. Am. Mosq. Control Assoc. 36, 152–160. 10.2987/20-69571 33600583

[B2] Arias-Goeta C. Mousson L. Rougeon F. Failloux A.-B. (2013). Dissemination and Transmission of the E1-226V Variant of Chikungunya Virus in *Aedes albopictus* Are Controlled at the Midgut Barrier Level. PLoS One 8 (2), e57548. 10.1371/journal.pone.0057548 23437397 PMC3578806

[B3] Armstrong P. M. Ehrlich H. Y. Magalhaes T. Miller M. R. Conway P. J. Bransfield A. (2020). Successive Blood Meals Enhance Virus Dissemination within Mosquitoes and Increase Transmission Potential. Nat. Microbiol. 5, 239–247. 10.1038/s41564-019-0619-y 31819213 PMC7199921

[B4] Bakri A. Mehta K. Lance D. (20052005). “Sterilizing Insects with Ionizing Radiation,” in Sterile Insect Technique. Editors Dyck V. A. Hendrichs J. Robinson A. S. (Dordrecht, Netherlands: Springer), 233–268. 1-4020-4050-4.

[B5] Balestrino F. Medici A. Candini G. Carrieri M. Maccagnani B. Calvitti M. (2010). γ Ray Dosimetry and Mating Capacity Studies in the Laboratory on *Aedes albopictus* Males. J. Med. Entomol. 47, 581–591. 10.1093/jmedent/47.4.581 20695273 PMC7027263

[B6] Balestrino F. Puggioli A. Gilles J. R. L. Bellini R. (2014). Validation of a New Larval Rearing Unit for *Aedes albopictus* (Diptera: Culicidae) Mass Rearing. PLoS One 9, e91914. 10.1371/journal.pone.0091914 24647347 PMC3960149

[B7] Balestrino F. Mathis A. Lang S. Veronesi E. (2016). Sterilization of Hulecoeteomyia Japonica Japonica (= Aedes Japonicus Japonicus ) (Theobald, 1901) by High‐energy Photon Irradiation: Implications for a Sterile Insect Technique Approach in Europe. Med. Vet. Entomol. 30, 278–285. 10.1111/mve.12170 27091384

[B8] Balestrino F. Puggioli A. Carrieri M. Bouyer J. Bellini R. (2017). Quality Control Methods for *Aedes albopictus* Sterile Male Production. PLoS Negl. Trop. Dis. 11, e0005881. 10.1371/journal.pntd.0005881 28892483 PMC5608434

[B9] Bellini R. Albieri A. Balestrino F. Carrieri M. Porretta D. Urbanelli S. (2010). Dispersal and Survival of *Aedes albopictus* (Diptera: Culicidae) Males in Italian Urban Areas and Significance for Sterile Insect Technique Application. Jnl. Med. Entom. 47, 1082–1091. 10.1603/me09154 21175057

[B10] Bellini R. Medici A. Puggioli A. Balestrino F. Carrieri M. (2013). Pilot Field Trials with *Aedes albopictus* Irradiated Sterile Males in Italian Urban Areas. Jnl. Med. Entom. 50, 317–325. 10.1603/me12048 23540120

[B12] Benedict M. Q. Levine R. S. Hawley W. A. Lounibos L. P. (2007). Spread of the Tiger: Global Risk of Invasion by the MosquitoAedes Albopictus. Vector-Borne Zoonotic Dis. 7, 76–85. 10.1089/vbz.2006.0562 17417960 PMC2212601

[B13] Bond J. G. Osorio A. R. Avila N. Gómez-Simuta Y. Marina C. F. Fernández-Salas I. (2019). Optimization of Irradiation Dose to *Aedes aegypti* and Ae. Albopictus in a Sterile Insect Technique Program. PLoS One 14, e0212520. 10.1371/journal.pone.0212520 30779779 PMC6380561

[B15] Bouyer J. Yamada H. Pereira R. Bourtzis K. Vreysen M. J. B. (2020a). Phased Conditional Approach for Mosquito Management Using Sterile Insect Technique. Trends Parasitol. 36, 325–336. 10.1016/j.pt.2020.01.004 32035818

[B16] Brady O. J. Johansson M. A. Guerra C. A. Bhatt S. Golding N. Pigott D. M. (2013). Modelling Adult *Aedes aegypti* and *Aedes albopictus* Survival at Different Temperatures in Laboratory and Field Settings. Parasites Vectors 6, 351. 10.1186/1756-3305-6-351 24330720 PMC3867219

[B17] Burnham K. P. Anderson D. R. (2003). Model Selection and Multimodel Inference: A Practical Information-Theoretic Approach. New York: Springer Science & Business Media.

[B18] Cai Z. Yao Z. Li Y. Xi Z. Bourtzis K. Zhao Z. (2018). Intestinal Probiotics Restore the Ecological Fitness Decline of *Bactrocera Dorsalis* by Irradiation. Evol. Appl. 11, 1946–1963. 10.1111/eva.12698 30459840 PMC6231467

[B19] Christofferson R. C. Chisenhall D. M. Wearing H. J. Mores C. N. (2014). Chikungunya Viral Fitness Measures within the Vector and Subsequent Transmission Potential. PLoS One 9, e110538. 10.1371/journal.pone.0110538 25310016 PMC4195746

[B20] Culbert N. J. Balestrino F. Dor A. Herranz G. S. Yamada H. Wallner T. (2018). A Rapid Quality Control Test to Foster the Development of Genetic Control in Mosquitoes. Sci. Rep. 8, 16179. 10.1038/s41598-018-34469-6 30385841 PMC6212531

[B21] Cunningham C. A. Aldridge R. L. Kline J. Bibbs C. S. Linthicum K. J. Xue R. D. (2020). Effects of Radiation on Blood‐feeding Activity of *Aedes aegypti* (Diptera: Culicidae). J. Vector Ecol. 45, 140–141. 10.1111/jvec.12382 32492280

[B22] da Silveira I. D. Petersen M. T. Sylvestre G. Garcia G. A. David M. R. Pavan M. G. (2018). Zika Virus Infection Produces a Reduction on *Aedes aegypti* Lifespan but No Effects on Mosquito Fecundity and Oviposition Success. Front. Microbiol. 9, 3011. 10.3389/fmicb.2018.03011 30619118 PMC6305470

[B23] Dame D. A. Curtis C. F. Benedict M. Q. Robinson A. S. Knols B. G. (2009). Historical Applications of Induced Sterilisation in Field Populations of Mosquitoes. Malar. J. 8, S2. 10.1186/1475-2875-8-s2-s2 PMC2777324 19917072

[B24] Dennison N. J. Jupatanakul N. Dimopoulos G. (2014). The Mosquito Microbiota Influences Vector Competence for Human Pathogens. Curr. Opin. Insect Sci. 3, 6–13. 10.1016/j.cois.2014.07.004 25584199 PMC4288011

[B25] Dong S. Balaraman V. Kantor A. M. Lin J. Grant D. G. Held N. L. (2017). Chikungunya Virus Dissemination from the Midgut of *Aedes aegypti* Is Associated with Temporal Basal Lamina Degradation during Bloodmeal Digestion. PLoS Negl. Trop. Dis. 11, e0005976. 10.1371/journal.pntd.0005976 28961239 PMC5636170

[B26] Dubrulle M. Mousson L. Moutailler S. Vazeille M. Failloux A.-B. (2009). Chikungunya Virus and Aedes Mosquitoes: Saliva Is Infectious as Soon as Two Days after Oral Infection. PLoS One 4, e5895. 10.1371/journal.pone.0005895 19521520 PMC2690823

[B27] Dyck V. A. Hendrichs J. P. Robinson A. S. (Editors) (2021). The Sterile Insect Technique: Principles and Practice in Area-wide Integrated Pest Management. second edition (Boca Raton, US: CRC Press).

[B28] FAO (2005). in ISPM, Publication Number 5, International Plant Protection Convention (IPPC) (Rome, Italy: FAO).Glossary of Phytosanitary Terms

[B29] Gonçalves C. M. Melo F. F. Bezerra J. M. Chaves B. A. Silva B. M. Silva L. D. (2014). Distinct Variation in Vector Competence Among Nine Field Populations of *Aedes aegypti* from a Brazilian Dengue-Endemic Risk City. Parasites Vectors 7, 320. 10.1186/1756-3305-7-320 25015526 PMC4230638

[B30] Guissou E. Poda S. de Sales Hien D. F. Yerbanga S. R. Da D. F. Cohuet A. (2020). Effect of Irradiation on the Survival and Susceptibility of Female *Anopheles Arabiensis* to Natural Isolates of *Plasmodium Falciparum* . Parasites Vectors 13, 266. 10.1186/s13071-020-04135-w 32434542 PMC7238563

[B31] Guo X.-X. Zhu X.-J. Li C.-X. Dong Y.-D. Zhang Y.-M. Xing D. (2013). Vector Competence of *Aedes albopictus* and *Aedes aegypti* (Diptera: Culicidae) for DEN2-43 and New Guinea C Virus Strains of Dengue 2 Virus. Acta Trop. 128, 566–570. 10.1016/j.actatropica.2013.08.006 23962388

[B32] Hurvich C. M. Tsai C.-L. (1995). Model Selection for Extended Quasi-Likelihood Models in Small Samples. Biometrics 51, 1077–1084. 10.2307/2533006 7548692

[B33] IAEA (2012). Developing Alternatives to Gamma Irradiation for the Sterile Insect Technique. Nucl. Technol. Rev. 81-91.

[B34] Janeh M. Osman D. Kambris Z. (2017). Damage-Induced Cell Regeneration in the Midgut of *Aedes albopictus* Mosquitoes. Sci. Rep. 7, 44594. 10.1038/srep44594 28300181 PMC5353711

[B35] Janeh M. Osman D. Kambris Z. (2019). Comparative Analysis of Midgut Regeneration Capacity and Resistance to Oral Infection in Three Disease-Vector Mosquitoes. Sci. Rep. 9, 14556. 10.1038/s41598-019-50994-4 31601867 PMC6787257

[B36] Lauzon C. R. Potter S. E. (2012). Description of the Irradiated and Nonirradiated Midgut of *Ceratitis Capitata* Wiedemann (Diptera: Tephritidae) and *Anastrepha Ludens* Loew (Diptera: Tephritidae) Used for Sterile Insect Technique. J. Pest Sci. 85, 217–226. 10.1007/s10340-011-0410-1

[B37] Maciel-de-Freitas R. Koella J. C. Lourenço-de-Oliveira R. (2011). Lower Survival Rate, Longevity and Fecundity of *Aedes aegypti* (Diptera: Culicidae) Females Orally Challenged with Dengue Virus Serotype 2. Trans. R. Soc. Trop. Med. Hyg. 105, 452–458. 10.1016/j.trstmh.2011.05.006 21700303

[B38] Medlock J. M. Hansford K. M. Schaffner F. Versteirt V. Hendrickx G. Zeller H. (2012). A Review of the Invasive Mosquitoes in Europe: Ecology, Public Health Risks, and Control Options. Vector-Borne Zoonotic Dis. 12, 435–447. 10.1089/vbz.2011.0814 22448724 PMC3366101

[B39] Merwaiss F. Filomatori C. V. Susuki Y. Bardossy E. S. Alvarez D. E. Saleh M. C. (2021). Chikungunya Virus Replication Rate Determines the Capacity of Crossing Tissue Barriers in Mosquitoes. J. Virol. 95, e01956–20. 10.1128/JVI.01956-20 33148794 PMC7925089

[B40] Mousson L. Martin E. Zouache K. Madec Y. Mavingui P. Failloux A. B. (2010). Wolbachiamodulates Chikungunya Replication inAedes Albopictus. Mol. Ecol. 19, 1953–1964. 10.1111/j.1365-294x.2010.04606.x 20345686

[B41] Mousson L. Zouache K. Arias-Goeta C. Raquin V. Mavingui P. Failloux A.-B. (2012). The Native Wolbachia Symbionts Limit Transmission of Dengue Virus in *Aedes albopictus* . PLoS Negl. Trop. Dis. 6, e1989. 10.1371/journal.pntd.0001989 23301109 PMC3531523

[B42] Muir L. E. Kay B. H. (1998). *Aedes aegypti* Survival and Dispersal Estimated by Mark-Release-Recapture in Northern Australia. Am. J. Trop. Med. Hyg. 58, 277–282. 10.4269/ajtmh.1998.58.277 9546403

[B43] Oliva C. F. Benedict M. Q. Collins C. M. Baldet T. Bellini R. Bossin H. (2021). Sterile Insect Technique (SIT) against Aedes Species Mosquitoes: a Roadmap and Good Practice Framework for Designing, Implementing and Evaluating Pilot Field Trials. Insects 12, 191. 10.3390/insects12030191 33668374 PMC7996155

[B44] O'Reilly D. R. Miller L. K. Luckow V. A. (1994). Baculovirus Expression Vectors: A Laboratory Manual. New York: Oxford University Press.

[B45] Parker A. G. Vreysen M. J. B. Bouyer J. Calkins C. O. (2021). “Sterile Insect Quality Control/assurance,” in Sterile Insect Technique. Principles and Practice in Area-wide Integrated Pest Management. Editors Dyck V. A. Hendrichs J. Robinson A. S.. 2 nd edition (Boca Raton, FL, US: CRC Press), 399–440. 10.1201/9781003035572-12

[B46] Paupy C. Delatte H. Bagny L. Corbel V. Fontenille D. (2009). *Aedes albopictus*, an Arbovirus Vector: from the Darkness to the Light. Microbes Infect. 11, 1177–1185. 10.1016/j.micinf.2009.05.005 19450706

[B47] Richards S. L. Anderson S. L. Smartt C. T. (2010). Vector Competence of Florida Mosquitoes for Chikungunya Virus. J. Vector Ecol. 35, 439–443. 10.1111/j.1948-7134.2010.00105.x 21175954 PMC3076135

[B48] Schaffner F. Mathis A. (2014). Dengue and Dengue Vectors in the WHO European Region: Past, Present, and Scenarios for the Future. Lancet Infect. Dis. 14, 1271–1280. 10.1016/s1473-3099(14)70834-5 25172160

[B49] Schaffner F. Medlock J. M. Bortel W. V. (2013). Public Health Significance of Invasive Mosquitoes in Europe. Clin. Microbiol. Infect. 19, 685–692. 10.1111/1469-0691.12189 23574618

[B50] Schuffenecker I. Iteman I. Michault A. Murri S. Frangeul L. Vaney M.-C. (2006). Genome Microevolution of Chikungunya Viruses Causing the Indian Ocean Outbreak. PLoS Med. 3, e263. 10.1371/journal.pmed.0030263 16700631 PMC1463904

[B51] Taracena M. L. Bottino-Rojas V. Talyuli O. A. C. Walter-Nuno A. B. Oliveira J. H. M. Angleró-Rodriguez Y. I. (2018). Regulation of Midgut Cell Proliferation Impacts *Aedes aegypti* Susceptibility to Dengue Virus. PLoS Negl. Trop. Dis. 12, e0006498. 10.1371/journal.pntd.0006498 29782512 PMC5983868

[B52] Tjaden N. B. Thomas S. M. Fischer D. Beierkuhnlein C. (2013). Extrinsic Incubation Period of Dengue: Knowledge, Backlog, and Applications of Temperature Dependence. PLoS Negl. Trop. Dis. 7, e2207. 10.1371/journal.pntd.0002207 23826399 PMC3694834

[B53] Vavassori L. Saddler A. Müller P. (2019). Active Dispersal of *Aedes albopictus*: a Mark-Release-Recapture Study Using Self-Marking Units. Parasites Vectors 12, 583. 10.1186/s13071-019-3837-5 31831040 PMC6909613

[B54] Vazeille-Falcoz M. Rodhain F. Chungue E. Failloux A. B. Mousson L. (1999). Variation in Oral Susceptibility to Dengue Type 2 Virus of Populations of *Aedes aegypti* from the Islands of Tahiti and Moorea, French Polynesia. Am. J. Trop. Med. Hyg. 60, 292–299. 10.4269/ajtmh.1999.60.292 10072154

[B55] Vazeille M. Moutailler S. Coudrier D. Rousseaux C. Khun H. Huerre M. (2007). Two Chikungunya Isolates from the Outbreak of La Reunion (Indian Ocean) Exhibit Different Patterns of Infection in the Mosquito, *Aedes albopictus* . PLoS One 2, e1168. 10.1371/journal.pone.0001168 18000540 PMC2064959

[B56] Vazeille M. Mousson L. Martin E. Failloux A.-B. (2010). Orally Co-infected *Aedes albopictus* from La Reunion Island, Indian Ocean, Can Deliver Both Dengue and Chikungunya Infectious Viral Particles in Their Saliva. PLoS Negl. Trop. Dis. 4, e706. 10.1371/journal.pntd.0000706 20544013 PMC2882319

[B57] Vega-Rúa A. Zouache K. Girod R. Failloux A.-B. Lourenço-de-Oliveira R. (2014). High Level of Vector Competence of *Aedes aegypti* and *Aedes albopictus* from Ten American Countries as a Crucial Factor in the Spread of Chikungunya Virus. J. Virol. 88, 6294–6306. 10.1128/jvi.00370-14 24672026 PMC4093877

[B58] WHO/IAEA (2020). Guidance Framework for Testing the Sterile Insect Technique (SIT) as a Vector Control Tool against Aedes-Borne Diseases. Geneva: World Health Organization and the International Atomic Energy Agency. pp190.

[B59] Wilson A. L. Courtenay O. Kelly-Hope L. A. Scott T. W. Takken W. Torr S. J. (2020). The Importance of Vector Control for the Control and Elimination of Vector-Borne Diseases. PLoS Negl. Trop. Dis. 14, e0007831. 10.1371/journal.pntd.0007831 31945061 PMC6964823

[B60] Woruba D. N. Morrow J. L. Reynolds O. L. Chapman T. A. Collins D. P. Riegler M. (2019). Diet and Irradiation Effects on the Bacterial Community Composition and Structure in the Gut of Domesticated Teneral and Mature Queensland Fruit Fly, *Bactrocera Tryoni* (Diptera: Tephritidae). BMC Microbiol. 19 (S1), 281. 10.1186/s12866-019-1649-6 31870300 PMC6929413

